# Metabolomic Analysis of Alfalfa (*Medicago sativa* L.) Root-Symbiotic Rhizobia Responses under Alkali Stress

**DOI:** 10.3389/fpls.2017.01208

**Published:** 2017-07-11

**Authors:** Tingting Song, Huihui Xu, Na Sun, Liu Jiang, Pu Tian, Yueyuan Yong, Weiwei Yang, Hua Cai, Guowen Cui

**Affiliations:** ^1^College of Animal Sciences and Technology, Northeast Agricultural University Harbin, China; ^2^College of Life Sciences, Northeast Agricultural University Harbin, China

**Keywords:** alfalfa, alkali stress, symbiotic rhizobium, metabolomics, GC-TOF/MS

## Abstract

Alkaline salts (e.g., NaHCO_3_ and Na_2_CO_3_) causes more severe morphological and physiological damage to plants than neutral salts (e.g., NaCl and Na_2_SO_4_) due to differences in pH. The mechanism by which plants respond to alkali stress is not fully understood, especially in plants having symbotic relationships such as alfalfa (*Medicago sativa* L.). Therefore, a study was designed to evaluate the metabolic response of the root-nodule symbiosis in alfalfa under alkali stress using comparative metabolomics. Rhizobium-nodulized (RI group) and non-nodulized (NI group) alfalfa roots were treated with 200 mmol/L NaHCO_3_ and, roots samples were analyzed for malondialdehydyde (MDA), proline, glutathione (GSH), superoxide dismutase (SOD), and peroxidase (POD) content. Additionally, metabolite profiling was conducted using gas chromatography combined with time-of-flight mass spectrometry (GC/TOF-MS). Phenotypically, the RI alfalfa exhibited a greater resistance to alkali stress than the NI plants examined. Physiological analysis and metabolic profiling revealed that RI plants accumulated more antioxidants (SOD, POD, GSH), osmolytes (sugar, glycols, proline), organic acids (succinic acid, fumaric acid, and alpha-ketoglutaric acid), and metabolites that are involved in nitrogen fixation. Our pairwise metabolomics comparisons revealed that RI alfalfa plants exhibited a distinct metabolic profile associated with alkali putative tolerance relative to NI alfalfa plants. Data provide new information about the relationship between non-nodulized, rhizobium-nodulized alfalfa and alkali resistance.

## Introduction

Alfalfa (*Medicago sativa* L.) is an important forage legume that is cultivated widely throughout the world (Chao et al., 2009). The USA is the largest producer of alfalfa ranking fourth behind corn, wheat, and soybean among all cultivated crops (Fernandez-Cornejo et al., 2016). However, the worsening of the global environment and increasing soil salinization have diminished the quality and yields of alfalfa grass (Wang et al., 2001).

Soils may be salinized by neutral salts (NaCl and Na_2_SO_4_) or alkaline salts (NaHCO_3_ and Na_2_CO_3_), with the latter form causing alkali stress, which is particularly detrimental to plants (Yang et al., 2007). Injury caused by salt stress is often attributed to low water potential and ion toxicity. Alkaline salts, in addition to imposing these two stresses, also cause high-pH stress (Yang et al., 2008b, 2009). High pH in the rhizosphere disrupts the physiological functions of the root and under severe condition can even destroy the membrane structures of roots. High pH also disturbs the homeostasis of various mineral ions. Hence, encompassing both salt and pH stressors, alkali stress effects tend to be more pronounced than ion poisoning or osmotic stress. The markedly lower tolerance of plants to alkali (alkaline salt) stress than to salinity (neutral salt) stress has been reported (Yang et al., 2008a; Girvin, 2010; Wang et al., 2012).

Many studies have studied the mechanisms of stress tolerance in model plants such as *Arabidopsis thaliana, Oryza sativa*, and *Lotus corniculatus* L. Plants have evolved complex mechanisms involving a series of gene expression and gene product interactions that enable them to adapt to abiotic stress at both the cellular and molecular level (Jin et al., 2006). A number of stress response genes have been identified in plants, including ion transporters, free radical scavengers, aquaporin ns, heat shock proteins, and late embryogenesis abundant proteins (Wang et al., 2003). Plants cope with alkali stress by regulating intracellular pH to re-establish ionic balance and by adjusting the pH of their rhizospheres, which is costly in terms of energy and resources (Guo et al., 2015).

*Medicago truncatula* is a model or reference species for legume genetics, genomics, and breeding. Examination of plant salt stress responses in the *Medicago truncatula* have focused primarily on basal metabolism, photosynthesis, ion content changes under salt stress, and salt tolerance gene cloning and transformation (Zhuo et al., 2013; Sun et al., 2014; Tang et al., 2014). Salt resistance is also being investigated in alfalfa with high-throughput genome sequencing and multiple-omics strategies (i.e., transcriptomics, proteomics and metabolomics) (Alvarez et al., 2008; Guo et al., 2015). Such studies have screened for differentially expressed genes, proteins, and metabolites with the aim of elucidating the physiological and molecular mechanisms of salt stress responses in alfalfa.

It is reasonable to hypothesize that many aspects of plant responses to alkali stress would be similar to salinity stress responses, such as ion transportation, osmotic solute accumulation, photosynthesis, and hormone synthesis (Guo et al., 2015). Metabolic solutes, such as proline, betaine, soluble sugar, polyamine, and polyols, have been shown to exhibit marked changes under salt stress, making them likely potential mediators of plant resistance to alkali stress (Munns and Tester, 2008; Guo et al., 2015). Nonetheless, the specific mechanisms by which plants respond to alkali stress are largely unknown and alkali-related metabolomics information remains limited.

The alfalfa can establish symbiosis with *Rhizobium* microbes, a family of gram-negative bacteria that are commonly found in soils (Denison and Kiers, 2011). In the presence of rhizobia, alfalfa roots undergo nodulation, establishing a symbiotic partnership wherein plants provide photosynthate and carbon resources to the rhizobia (Jones et al., 2007). In return, the nodulated rhizobia conduct nitrogen fixation by nitrogenase, thereby providing plants with usable nitrogen. In agriculture, *Rhizobium* nodulation not only reduces the need for nitrogen fertilizer, but can also improve the host plant's resistance to adverse conditions (Antolín and Sánchez-Díaz, 1992; Figueiredo et al., 2008; Yang et al., 2013; Wang et al., 2016b). Photosynthates are transported through the phloem to the nodule where sugar molecules are converted into dicarboxylic acid, mainly in the form of malic acid and succinic acid salt (Poynard et al., 2003). There are many intermediate metabolites during this symbiotic process, such as soluble polysaccharides, organic acids, and amino acids, which play positive roles in plant's regulation of cell osmotic pressure, pH balance, and reactive oxygen species (ROS) removal. Under adverse conditions, other nodule functions in nitrogen-fixing (i.e., carbohydrate utilization, nitrogen-containing compound accumulation, O_2_ permeability, and ROS accumulation) are inhibited (Arrese-Igor, 1999; Naya and Becana, 2007; Becana et al., 2010; Aranjuelo et al., 2011, 2013). Although nitrogen fixation in alfalfa and stress resistance in both alfalfa and rhizobia have been intensively studied, the mechanism through which rhizobia-alfalfa symbiosis supports alkali stress tolerance is unclear.

Therefore, the aim is to demonstrate the possible difference between NI and RI group under alkali stress in physiology and metabolic level, then deduce the response mechanisms of nodulized alfalfa under alkali stress at physiological and metabolomics levels. First, we compared alkali stress resistance between nodulized and non-nodulized alfalfa plants in physiologic level, and, then assayed nodule metabolites using gas chromatography combined with time-of-flight mass spectrometry (GC-TOF/MS) analysis to explore the mechanisms of nodule symbiosis in alkali resistance in metabolic level. Our study identified many important metabolites and metabolic pathways that participate in alkali resistance in alfalfa. Based on the results, we could deduce the key enzymes and genes in the enriched metabolic pathway. These results combined with physiological and metabolic level, contributes to a better understanding of the relationship between root-symbiotic rhizobia and alkali resistance.

## Materials and methods

### Plant materials and alkali treatment

*Medicago sativa* (Longmu 806) seeds were surface-sterilized with 75% ethanol for 15 s and 10% sodium hypochlorite for 5 min, and then rinsed thoroughly four or five times with sterile water. The seeds were germinated on wet filter paper in Petri dishes in a light growth chamber (light 24°C/dark 18°C, 16 h/8 h) and kept moist during the germination period (relative humidity, 60%). After germinating for 5 d, the normal plants were transplanted directly into a culture pot of sandy soil; nodule plants were transplanted into the sand culture bowl after inoculation with rhizobia inoculation nodules. Each tray was loaded with 24 pots, and each pot held three plants (H14 cm × D 13 cm). The plants were maintained in a greenhouse for 12 weeks (with 30°C/25°C day/night temperatures, 16 h of light and 40% relative humidity. Plants were irrigated twice weekly with different nutrient solutions for NI and RI samples. Normal plants were watered with 1/5 Hoagland nutrient solution. Plants with symbiotic rhizobium were watered with 1/5 Hoagland nutrient solution for the first 8 weeks and then 1/5 nitrogen-free nutrient solution for the subsequent 4 weeks to trigger nodule activation. After 12 weeks, control normal plants and control inoculating rhizobia alfalfa plants were maintained with 1/5 Hoagland nutrient solution and 1/5 nitrogen-free nutrient solution, respectively. Meanwhile, treated plants were submersed in the nutrient solution supplemented with 200 mmol/L NaHCO3 (Zhu et al., 2014; Jia et al., 2015). The treatment continued for 8 d, root tissues were collected on day 0 and day 6 after administration of alkali treatment. Roots were harvested by briefly rinsing away sand and dissecting out all of the root tissues (including nodules). The harvested root samples were frozen in liquid nitrogen. Treated and control plants were harvested at the same time. Twelve plants in each group (alkali stress and control group for each time point and each accession) served as the sample sources for detection of physiological compound; six plants from each time point served as the sample sources for metabolite extraction and metabolite profiling analysis.

### Detection of physiological indices

Malondialdehyde (MDA) content was determined according to a modified thiobarbituric acid (TBA) method (Puckette et al., 2007). Root tissue samples (200 mg) were homogenized in 5 mL of 0.1% (w/v) TCA, centrifuged at 10,000 × g for 5 min. Four milliliters of TCA (20%)/TBA (0.5%) solution was to a 1-ml aliquot of the supernatent. The mixture was heated to 100°C for 15 min, cooled on ice, and then centrifuged at 10,000 × g for 10 min. The absorbance of the supernatant was measured at 450, 532, and 600 nm. MDA concentration served as an index of lipid peroxidation.

Proline content was measured by the sulfosalicylic acid reaction method as described (Wang et al., 2016a). Root tissue samples were each homogenized in 10 mL of 3% (w/v) sulfosalicylic acid, and the homogenate was centrifuged at 3,000 × g for 20 min. Two milliliters of the supernatant was mixed with 2 mL of glacial acetic acid and 2 mL of acidic ninhydrin reagent, and then boiled for 40 min. The tubes were cooled, 5 mL of toluene was added to each tube, and absorbance at 520 nm was determined.

Superoxide dismutase (SOD) activity was measured by the nitroblue tetrazoliun (NBT) method (Giannopolitis and Ries, 1977; Yang et al., 2013). One unit of SOD activity was defined as the amount of enzyme required to inhibit the reduction rate of NBT by 50%. Peroxidase (POD) activity was measured by determining the oxidation rate of guaiacol (1-hydroxy-2-methoxybenzene, C_7_H_8_O_2_) substrate per minute (Yang et al., 2013).

Glutathione (GSH) reduction was assessed fluorometrically (Hissin and Hilf, 1976). Root samples were ground in 3 mL of 5% H_3_PO_3_. The homogenate was centrifuged at 13,000 × g for 10 min. The reaction mixture (3 mL), containing 2.6 mL of 150 mM PBS (pH 7.0), 0.2 mL of 0.1 mM dithiobis-2-nitrobenzoic acid (DTNB), and 0.2 mL of supernatant, was incubated at 30°C for 5 min and absorbance at 412 nm was recorded.

### Metabolite extraction and metabolite profiling analysis

The GC-MS was performed as described by Lisec et al. (2006). Briefly, 0.05 g aliquots of each sample were placed in 2-ml EP tubes. Metabolites were extracted from each tube with 0.4 ml of methanol-water (v/v = 3:1); 20 μl of Adonitol (0.2 mg/ml stock in ddH2O) was added as an internal standard. The mixture was vortexed for 30 s, homogenized in a ball mill for 3 min at 65 Hz, and then centrifuged at 12,000 rpm for 15 min at 4°C. The resultant supernatant (~0.35 ml) was transferred to a new 2-ml GC/MS glass vial; equal volumes of each sample (14 μl) were combined in a separate 2-ml GC/MS glass vial to serve as a mixed sample for quality control. The extracts were dried in a vacuum concentrator (37°C) for 2 h, and then 40 μl of methoxylamine hydrochloride (dissolved in pyridine, final concentration of 20 mg/ml) was added to each dried metabolite sample. The extracts were incubated at 80°C for 20 min in an oven after mixing and sealing. The lid of each tube was opened and 50 μl of BSTFA (containing 1% TCMS, v/v) was added to each sample, and then the tubes were re-sealed and incubated for 1 h at 70°C. Subsequently, 5 μl of FAMEs (standard mixture of fatty acid methyl esters, C8–C16: 1 mg/ml; C18–C24: 0.5 mg/ml in chloroform) was added to each mixed sample. The samples were allowed to cool to room temperature, and then mixed well in preparation for GC-TOF-MS analysis.

GC-TOF-MS analysis was performed with an Agilent 7890 gas chromatograph system (Agilent, USA) coupled with a Pegasus HT time-of-flight mass spectrometer. The system utilized a Rxi-5Sil MS column (30 m × 250 μm inner diameter, 0.25 μm film thickness; Restek, USA). A 1-μL aliquot of the analyte was injected in splitless mode. Helium was used as the carrier gas, the front inlet purge flow was 3mL·min^−1^, and the gas flow rate through the column was 1 mL·min^−1^. The initial temperature was kept at 50°C for 1 min, raised to 330°C at a rate of 10°C·min^−1^, and then kept for 5 min at 330°C. The injection, transfer line, and ion source temperatures were 280°C, 280°C, and 250°C, respectively. The energy level applied was −70 eV (electron impact mode). The mass spectrometry data were acquired in full-scan mode with an m/z range of 30–600 at a rate of 20 spectra/s after a solvent delay of 366 s.

Chroma TOF4.3X software (LECO Corporation) and the LECO-Fiehn Rtx5 database were used for raw peak extraction, data baseline filtering, baseline calibration, peak alignment, deconvolution analysis, peak identification, and integration of the peak area. The retention time index method was used for peak identification, and the retention time index tolerance was 5,000. For the GC-Quad FiehnLib library, derivatives were identified by increasing numbers according to retention index (e.g., serine 1, serine 2, and serine 3) for derivatives with one or two trimethylsilyl groups in the primary amino group a (Sumner et al., 2007; Kind et al., 2009; Creek et al., 2014).

### Data analysis and metabolic pathway construction

Determination of physiological indices were statistically analyzed using SPSS20.0. To reveal significant differences, the data were subjected to analyses of variance (ANOVAs), and the least significant differences (LSDs) of means were determined with Duncan's test (significance criterion, α = 0.05).

The resulting three-dimensional data, including peak number, sample name, and normalized data were imported into SIMCA software (V14, Umetrics AB, Umea, Sweden), principal component analysis (PCA) and orthogonal projections to latent structure-discriminant analysis (OPLS-DA) models were tested for all samples. The OPLS-DA model was employed with first principal-component of VIP (variable importance in the projection) values (VIP > 1) combined with Student's *T*-test (*T*-test) (*P* < 0.05) to find differentially accumulated/depleted metabolites; non-commercial databases, including KEGG (http://www.genome.jp/kegg/) and NIST (http://www.nist.gov/index.html), were searched for metabolite pathways. Quantitative normalization within replicates were subjected to logarithmic base 2 transformation. MetaboAnalyst online analysis software (www.metaboanalyst.ca/) was used to build heatmap diagrams (Xia and Wishart, 2011). Metabolomics data have been deposited to the EMBL-EBI MetaboLights database (Haug et al., 2013) with the identifier MTBLS481. The complete dataset can be accessed here http://www.ebi.ac.uk/metabolights/MTBLS481.

## Results

### Phenotype of alfalfa under alkali stress

**Figure 1:**
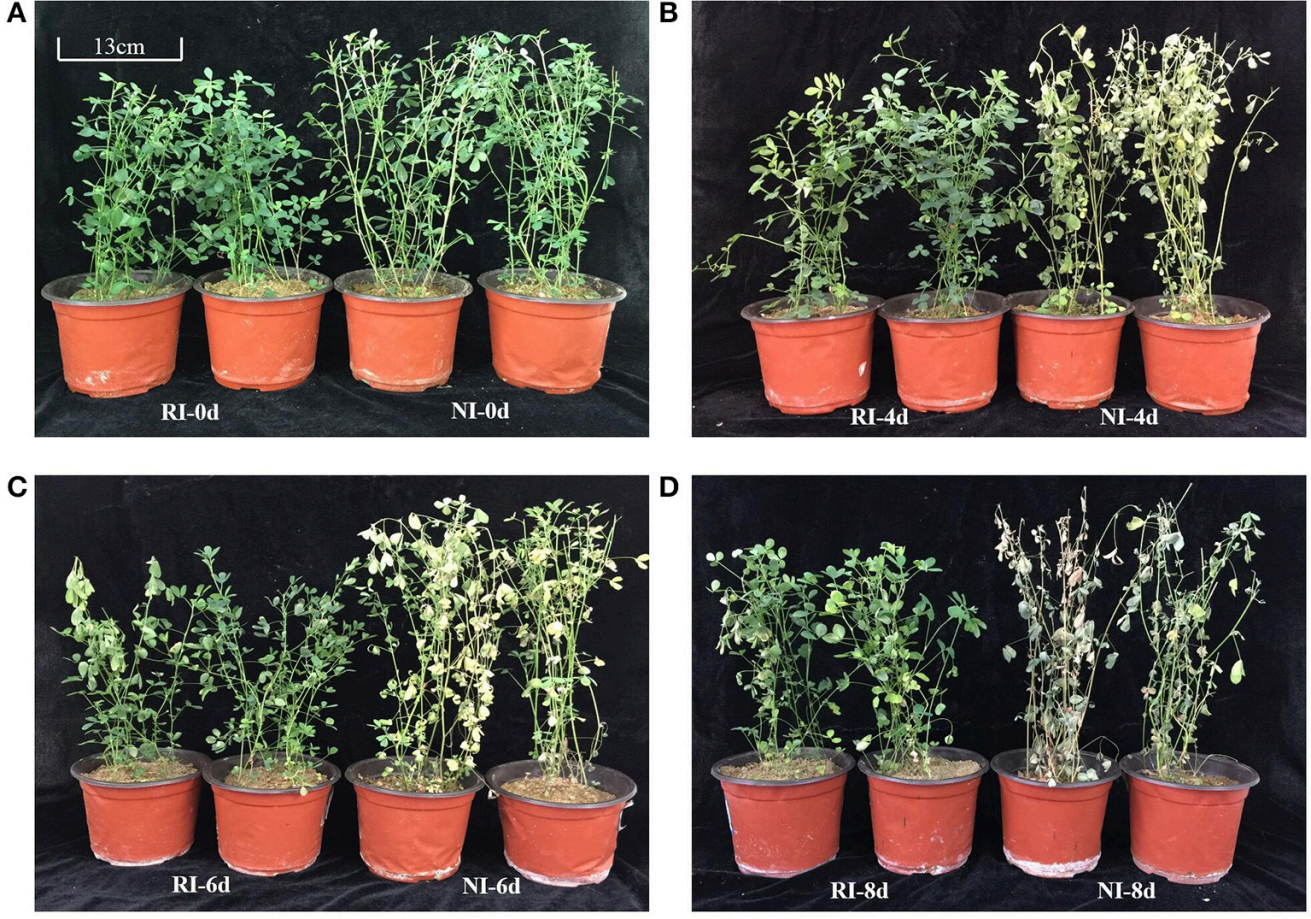
Phenotype of alfalfa plants with or without rhizobium inoculation before and after NaHCO_3_ treatment. **(A)** Untreated, **(B)** 4 d after alkali stress, **(C)** 6 d after alkali stress, and **(D)** 8 d after alkali stress. The left two pots in each image have inoculated RI group plants and the right two are non-inoculated NI plants.

Four days after the 200 mmol/L NaHCO_3_ treatment, leaves of alfalfa plants in the NI (no rhizobium inoculation) condition started to wilt and turned yellow, while no apparent damage was observed in the RI (nodulized with rhizobium) group (Figure 1B). Six days after treatment, the growth of plants in both groups was suppressed with the leaves of NI plants exhibiting serious wilting, while old leaves on RI plants begun to show some wilting (Figure 1C). Eight days after treatment, NI plants were dying while RI plants showed only limited damage (Figure 1D). The results above showed that phenotypically, rhizobium nodulation alleviated the damage induced by alkali stress and increased alkali tolerance in alfalfa (Figure 1).

### Physiological changes in alfalfa root under alkali stress

**Figure 2:**
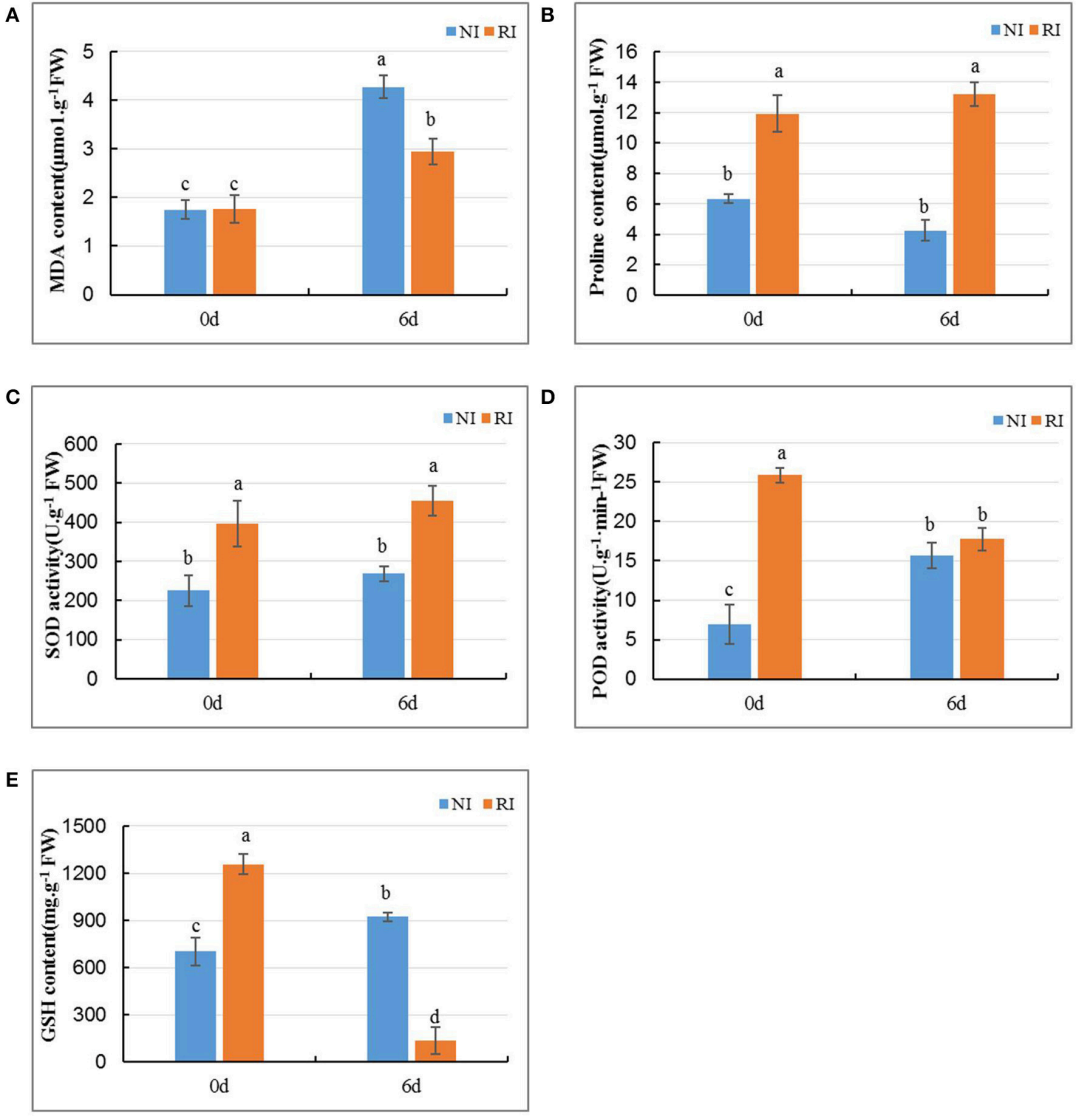
Changes in MDA **(A)** and proline **(B)** and GSH **(E)** content and in the activities of SOD **(C)**, and POD **(D)** in the roots of alfalfa plants with (RI group) and without (NI group) rhizobium inoculation under alkali stress. Values are means of six replicates. Standard error bars annotated by different lower-case letters in the same stress type indicate significant differences at *p* < 0.05 according to Duncan's method.

No obvious morphologic differences were found among RI when exposed to 200 mmol/L NaHCO3 treatments for 4 days, while the plants were seriously hampered and grew abnormally when exposed to 6 days after treatment. Therefore, we selected the plant of 6 days after treatment for the physiological and metabolomics analysis. Levels of the membrane lipid peroxidation end product and damage marker malondialdehydyde (MDA) in NI and RI groups were similar before the 200 mmol/L NaHCO_3_ treatment (Figure 2A). Six days after 200 mmol/L NaHCO_3_ treatment, MDA levels were increased in both NI and RI groups, with levels being 1.45-fold higher in the NI group than in the RI group (*p* < 0.05), indicating that NI plants suffered more damage than RI plants.

The level of proline was 4.34-fold higher in the RI group than that in NI group (*p* < 0.005) even before the alkali treatment (Figure 2B). Six days after 200 mmol/L NaHCO_3_ treatment, proline levels had increased in the NI group relative to pretreatment levels (*p* < 0.05), whereas proline levels in the RI group remained similar to pretreatment levels.

SOD and POD are two major antioxidases that help plants reduce ROS levels and therefore damage by abiotic stress. Relative to that in the NI group, SOD activity levels in the RI group was 1.76-fold higher before the alkali stress treatment and 1.69-fold higher 6 days after alkali treatment (both *p* < 0.05) (Figure 2C). SOD activity levels in the NI group did not differ significantly before vs. 6 days after treatment. POD activity in the RI group was 3.7-fold higher than that in the NI group before treatment. POD activity in RI decreased 6 days post treatment (*p* < 0.05 vs. pretreatment), but remained slightly higher than NI, although not statistically significant (Figure 2D). Taken together, these data indicate that alfalfa plants in RI group had higher antioxidant activity than plants in the NI group.

Similar to SOD and POD, Glutathione (GSH) content was 1.79-fold higher in RI than in NI *(p* < 0.05) before alkali treatment (Figure 2E). However, unlike SOD and POD levels, GSH content in the RI group was lower than that in NI plants after 6 days of alkali treatment *(p* < 0.05). The higher activity of proline, SOD, POD, and GSH in RI than NI before alkali treatment indicate that rhizobium nodulation induces their expression.

### Metabolic profiling

**Figure 3:**
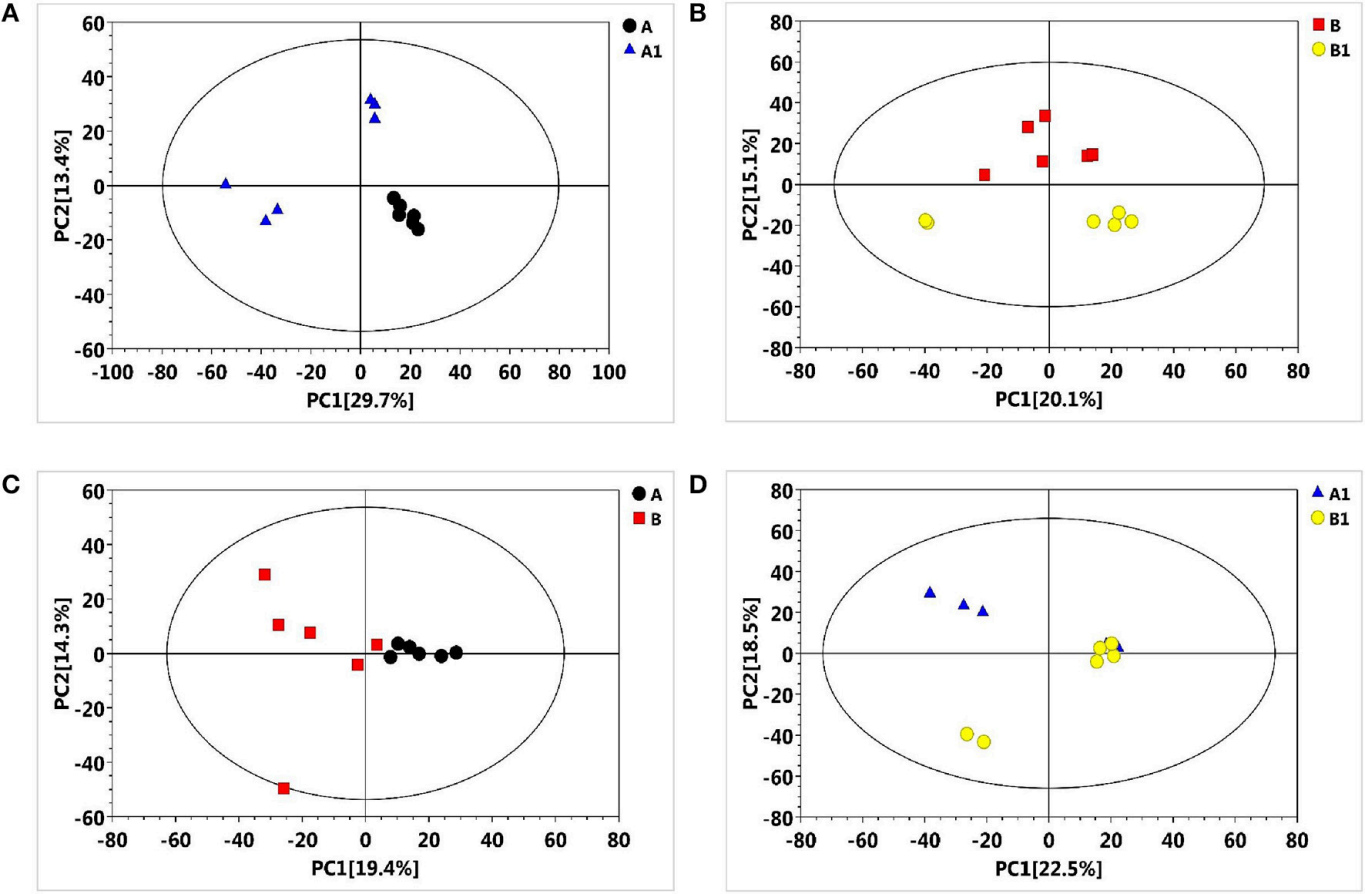
Principal component analysis (PCA) score plots of metabolic profiles in alfalfa roots under the alkali stress. **(A)** PCA score plot for NI control (black), NI alkali-treated (blue) samples, **(B)** PCA score plot for RI control (red) and RI alkali-treated (yellow) samples, **(C)** PCA score plot for NI control (black) and RI control (red) samples, **(D)** PCA score plot for NI alkali-treated (blue) and RI alkali-treated (yellow) samples.

**Figure 4:**
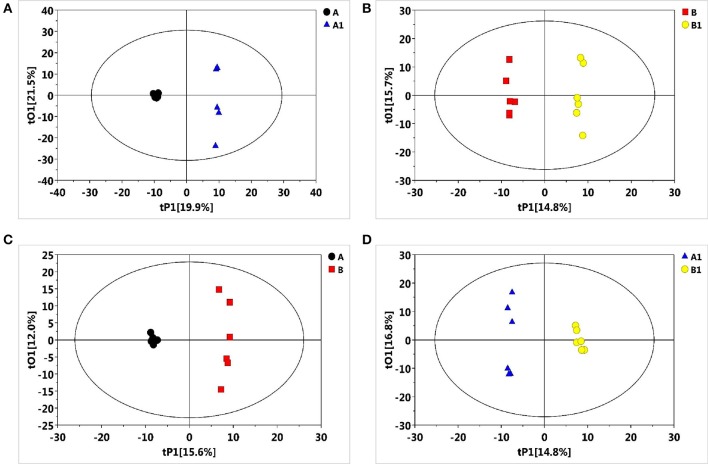
Partial least squares-discriminate analysis (PLS-DA) score plots of metabolic profiles in alfalfa roots under the alkali stress. **(A)** PLS-DA score plot for NI control (black), NI alkali-treated (blue) samples, **(B)** PLS-DA score plot for RI control (red) and RI alkali-treated (yellow) samples, **(C)** PLS-DA score plot for NI control (black) and RI control (red) samples, **(D)** PLS-DA score plot for NI alkali-treated (blue) and RI alkali-treated (yellow) samples.

To reveal the physiological responses and adaptive strategies of alfalfa to alkali stress, metabolic changes in NI and RI roots subjected to alkali treatment were assessed through GC/TOF-MS analysis. GC/TOF-MS analysis revealed an obvious chromatographic difference between the NI and RI groups. Using the interquartile range denoising method, we identified 483 metabolites from a total of 483 detected peaks. Then, missing values in the raw dataset were filled by half of the minimum value and the internal standard normalization method was applied. The resultant three-dimensional data, including peak numbers, sample names, and normalized peak areas, were entered into SIMCA14 software (Umetrics, Umea, Sweden) for principal component analysis (PCA) and orthogonal projections to latent structures-discriminate analysis (OPLS-DA) (Figures 3A–D, 4A–D). A PCA analysis score plot showed the distributions of the origin data and the samples were separated accordingly. However, the application of multivariate analysis OPLS-DA could be better to separate control from alkali stress samples and also separate no-rhizobium inoculation (NI) from rhizobium inoculation (RI) samples. The altered metabolites were found from the line plots of the X-loadings of the first component of the PLS-DA pairwise comparison models. And, the parameters for the classification indicated good predictability and goodness of fit (Figure S1). The variable importance in the projection (VIP) values greater than 1 and *p*-values lower 0.05 were considered the most relevant metabolites in response to alkali stress or symbiotic rhizobia.

### Metabolic changes in response to NI control, NI 6 d after alkali treatment

**Figure 5:**
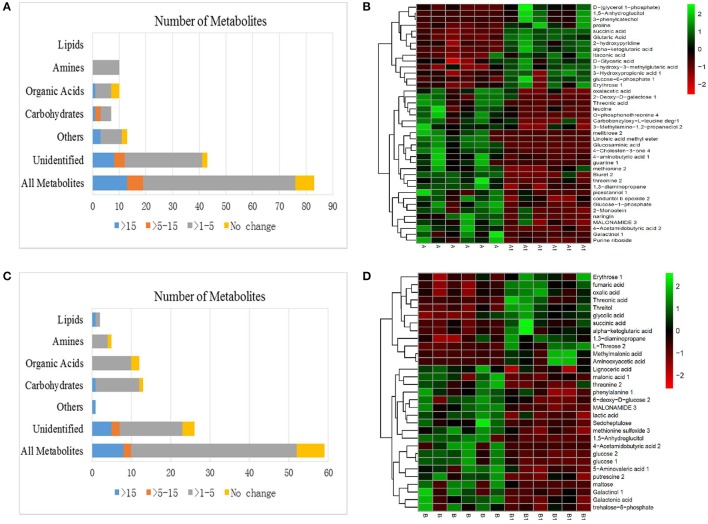
**(A)** Effects of alkali stress on the metabolomes of NI. **(B)** Heat map analysis combined with hierarchical cluster analysis of A and A1. **(C)** Effects of alkali stress on the metabolomes of RI. **(D)** Heat map analysis combined with hierarchical cluster analysis of B and B1. A, NI control; A1, NI 6 d after alkali stress; B, RI control; B1, RI 6 d after alkali stress.

A significant variance (VIP > 1, *p* < 0.05) in the metabolic profiles between NI control (A) and NI alkali treatment (A1) was observed and a total of 83 metabolites with significant differences were identified (Figure 5A). The 83 metabolites that were identified as differing between NI control and NI alkali stress, included carbohydrates, amines, organic acids, other polar molecules, and unidentified substances. Metabolites that showed a more than 15-fold difference (as calculated by the formula:${\text{log}}_{2}^{\left(A1/A\right)}$) before vs. after alkali treatment included melibiose (−18.00-fold), glucosaminic acid (−18.18-fold), 3-phenylcatechol (21.62-fold), and linoleic acid methyl ester (−24.13-fold); those with changes in the 5–15-fold ranged included galactinol (−5.22-fold), 1,5-anhydroglucitol (5.18-fold), and guanine (−6.46-fold). The rest of the metabolites examined, including various carbohydrates, amino acids, and organic acids, showed changes in the 1–5-fold range (Table 1 and Table S1). Heat map analysis showed the metabolites that exhibited significant changes in NI plants after alkali stress. Notably, osmolytes, such as proline, showed alkali-stress induced increases, furthermore, increases in ɑ-ketoglutarate and succinate, suggest up-regulation of the citric acid cycle (TCA). Meanwhile, the levels of some carbohydrates and organic amine substances were decreased in response to alkali stress (Figure 5B and Table 1).

**Table 1:** Relative concentration and fold changes of major metabolites in roots of NI and RI after 6 d of alkali treatment.

**Metabolite pathway**	**Metabolite**	**Relative concentration**				**Log fold change**			
		**NI**		**RI**					
		**Control (A)**	**Alkali (A1)**	**Control (B)**	**Alkali (B1)**	**${\text{Log}}_{2}^{\left(\text{A}1/\text{A}\right)}$**	**${\text{Log}}_{2}^{\left(\text{B}1/\text{B}\right)}$**	**${\text{Log}}_{2}^{\left(\text{B}/\text{A}\right)}$**	**${\text{Log}}_{2}^{\left(\text{B}1/\text{A}1\right)}$**
TCA cycle	Isocitric acid	1.85267	2.64897	1.24783	1.29777	0.51	0.05	−0.57	−1.03
	Succinic acid	0.36565	1.68136	0.12887	0.43829	2.20^**^	1.76^*^	−1.50^**^	−1.94^**^
	Fumaric acid	0.01884	0.02884	0.00903	0.02373	0.61	1.39^**^	−1.06^*^	−0.28
	Oxalacetic acid	0.00014	0.00003	0.00001	0.00004	−1.90^*^	1.22	−2.82^**^	0.30
	α**-**Ketoglutaric acid	0.00845	0.04659	0.00315	0.01164	2.46^**^	1.88^*^	−1.42^**^	−2.00^**^
Glycolysis	Glucose	0.44670	0.28891	0.45119	0.19332	−0.63	−1.22^**^	0.01	−0.58^**^
	Glucose-1-P	0.16496	0.04766	0.17287	0.14017	−1.79^*^	−0.30	0.07	1.56^*^
	Glucose-6-P	0.00047	0.00135	0.00129	0.00063	1.50^*^	−1.03	1.42	−1.10
Amino acids	Valine	0.03458	0.05676	0.05044	0.08056	0.71	0.67	0.54	0.51
	Serine	0.12241	0.24992	0.12479	0.16745	1.02	0.42	0.02	−0.58
	Leucine	0.01230	0.00193	0.01337	0.03904	−2.66^*^	1.54	0.12	4.34^*^
	Alanine	0.03461	0.03939	0.01152	0.02862	0.18	1.31	−1.58^*^	−0.46
	Glycine	0.02200	0.04539	0.00867	0.01671	1.04	0.95	−1.34^*^	−1.44
	Threonine	0.00691	0.00098	0.00844	0.00187	−2.80^**^	−2.17^*^	0.28	0.92
	Glutamine	0.00117	0.00105	0.00077	0.00110	−0.15	0.51	−0.60	0.07
	Asparagine	0.32631	0.39214	0.33561	0.12834	0.26	−1.38	0.04	−1.61
	Methionine	0.00016	0.00005	0.00005	0.00003	−1.46^*^	−0.64	−1.69^*^	−0.88
	Cycloleucine	0.02347	0.05551	0.02021	0.04270	1.24	1.07	−0.21	−0.38
	Proline	0.24178	2.01058	0.44890	1.37735	3.05^**^	1.61	0.89	−0.55
	GABA	0.50184	0.12516	0.54756	0.48122	−2.00^*^	−0.18	0.12	−16.95
	AOA	1.56E-09	0.00064	1.63E-09	0.00087	18.67	19.03^*^	0.07	0.44
Sugars and polyols	Fucose	0.00152	0.00057	0.00145	0.00163	−1.41	0.16	−0.06	1.51
	Fructose	0.09627	0.10910	0.15859	0.04860	0.18	−1.71	0.72	−1.17
	Maltose	0.04916	0.01935	0.09185	0.00809	−1.34	−3.51^*^	−0.90	−1.26
	Melibiose	0.00046	1.76E-09	0.00009	1.56E-09	−18.00^*^	−15.90	−2.26^*^	−0.17^*^
	Galactose	0.00044	0.00087	0.00064	0.00073	0.99	0.17	0.55	−0.27
	Ribose	0.10520	0.09000	0.13514	0.10425	−0.22	−0.37	0.36	0.21
	Lyxose	0.00029	0.00123	0.00025	0.00014	2.08	−0.82	−0.21	−3.12^*^
	Xylose	0.01907	0.02524	0.01439	0.01029	0.40	−0.48	−0.40	−1.29^*^
	Trehalose	0.00524	0.00428	1.63E-09	0.00267	−0.29	20.64	−21.61^*^	−0.68
	Erythrose	0.00058	0.00112	0.00066	0.00114	0.94^*^	0.78^*^	0.18	0.02
	Sophorose	0.00110	0.00343	0.00409	0.00337	1.64	−0.27	1.89	−0.03
	L-Threose	0.00029	0.00160	0.00022	0.00232	2.45	3.35^**^	−0.36	0.54
	6-Deoxy-D-glucose	1.55E-09	0.00009	0.00019	0.00004	15.97	−2.10^**^	16.96^**^	−1.12
	2-Deoxy-D-galactose	0.00167	0.00029	0.00056	0.00003	−2.49^*^	−4.19	−1.57	−3.28
	Ribitol	0.00022	0.00026	0.00020	0.00024	0.26	0.19	−0.08	−0.16
	Xylitol	0.00464	0.00514	0.00446	0.00213	0.14	−1.06	−0.05	−1.27
	Threitol	0.53763	0.26498	0.08333	0.31641	−1.02	1.92^**^	−2.68^**^	0.26
	Galactinol	0.00139	0.00003	0.00125	0.00016	−5.22^*^	−2.96^*^	−0.14	2.11
	1,5-Anhydroglucitol	0.00019	0.00723	0.00950	0.00052	5.18^*^	−4.18^**^	5.58^**^	−3.79^*^

*The relative concentration of each metabolite is an average of GC-TOF-MS data from six biological replicates*.

* *P < 0.05*,

** *P < 0.01. TCA, tricarboxylic acid; GABA, 4-acetamidobut-yric acid; AOA, aminooxyacetic acid*.

### Metabolic changes in response to RI control, RI 6 d after alkali treatment

As shown, 59 metabolites differed significantly between RI control (B) and RI alkali treatment (B1), including carbohydrates, amines, lipids, organic acids, other polar molecules, and unidentified molecules (Figure 5C). Metabolites that showed greater than 15-fold changes (as calculated by the formula:${\text{log}}_{2}^{\left(B1/B\right)}$) before vs. after the alkali treatment included 4-acetamidobutyric acid (−20.48-fold), and aminooxyacetic acid (19.03-fold). Metabolites showing differences between 1- and 5-fold included maltose (−3.51-fold), glucose (−1.22-fold), L-threose (3.36-fold-fold), threonine (−2.17-fold), putrescine (−1.72-fold), succinic acid (1.77-fold), fumaric acid (1.39-fold), threonic acid (2.58-fold), galactonic acid (−1.33-fold), and 1,3-diaminopropane (1.11-fold), among others (Table 1 and Table S1). Heat mapping showed distinct alkali-stress induced metabolite profile changes in the RI group relative to that in the NI group. Specifically, in the RI group, the alkali stress treatment resulted in more organic acid changes, such as, increases in ɑ-ketoglutarate, fumarate, and succinate, as well as and notable increases in the amino acid biosynthesis precursors. (Figure 5D and Table 1).

### Differences between NI and RI roots without alkali treatment

**Figure 6:**
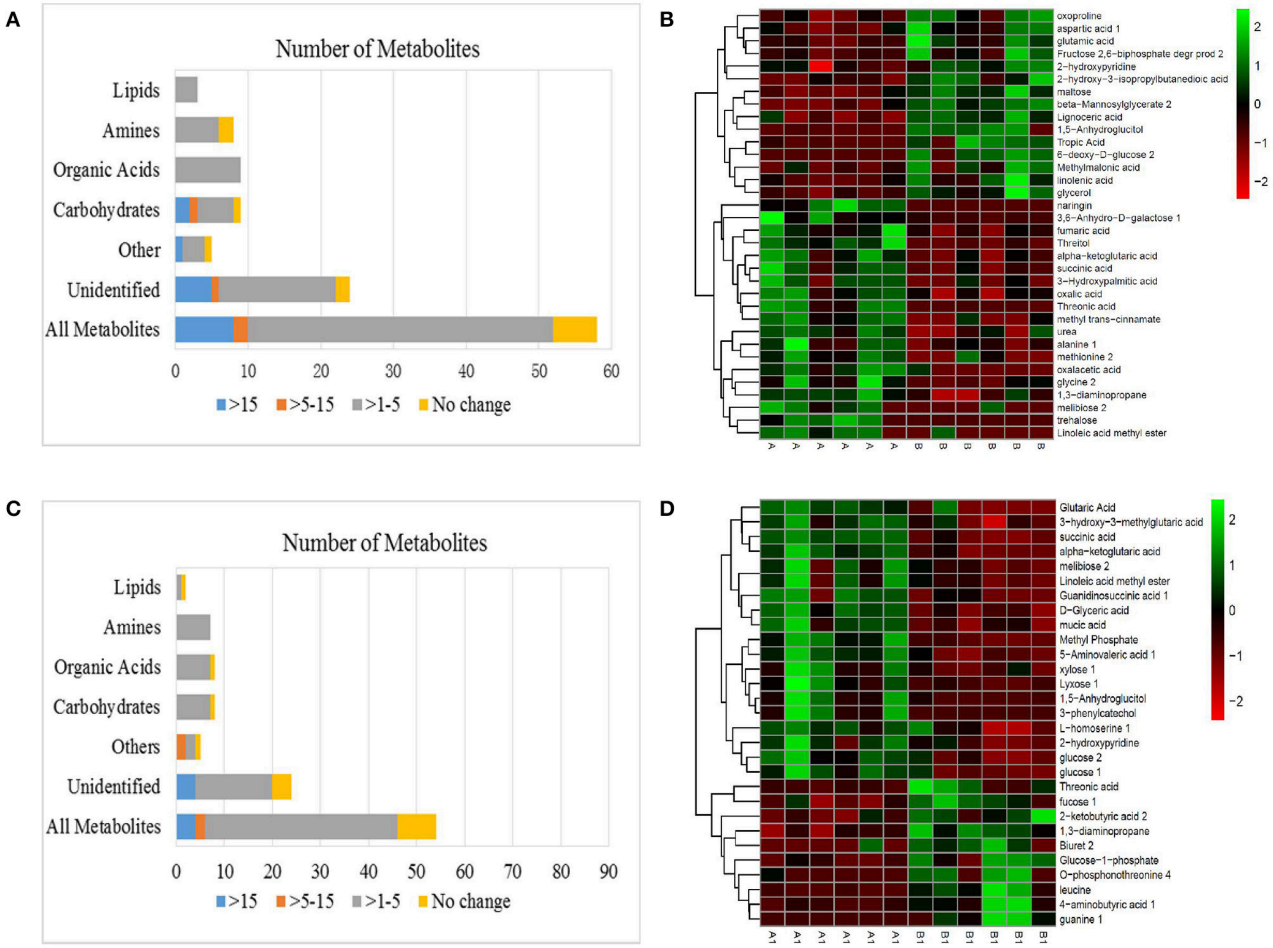
**(A)** Effects of metabolomes in NI and RI alfalfa. **(B)** Heat map analysis combined with hierarchical cluster analysis of A and B. **(C)** Effects of alkali stress on the metabolomes of NI and RI. **(D)** Heat map analysis combined with hierarchical cluster analysis of A1 and B1. A, NI control; B, RI control; A1, NI 6 d after alkali stress; B1, RI 6 d after alkali stress.

Fifty eight metabolite differences were identified between the NI and RI groups under normal conditions (Figure 6A). Metabolites with a greater than a 15-fold (as calculated by the formula:${\text{log}}_{2}^{\left(B1/A1\right)}$) difference between NI and RI included trehalose (−21.61-fold), 6-deoxy-D-glucose (16.96-fold), and naringin (−18.49-fold); only 1,5-anhydroglucitol (5.58-fold) had a difference in the 5- to 15-fold range. Noticeably, many organic acids and amines were 1- to 5-fold higher in NI plants than in RI plants (*p* < 0.05), including succinic acid (−1.50-fold), fumaric acid (−1.06-fold), threonic acid (−3.42-fold), ɑ-ketoglutaric acid (−1.42-fold), oxalic acid (−1.19-fold), glycine (−1.34-fold), alanine (−1.59-fold), urea (−1.41-fold), 1,3-diaminopropane (−1.27-fold), and methionine (−1.69-fold) (Table 1 and Table S1). Heat maps revealed distinctly different metabolite profiles for the NI and RI groups, even before the alkali stress treatment. Organic acids in the TCA cycle and most amines and carbohydrates were higher in NI than in RI plants (Figure 6B and Table 1).

### Differences between NI and RI roots under alkali treatment

A different but overlapping metabolite-response trend to NI (A1) and RI (B1) in alkali stress was determined in alfalfa. There were 53 different metabolites between NI and RI in alkali stress (Figure 6C). Among these metabolites, only 13 definite substances with fold < −1 (fold was calculated by the formula:${\text{log}}_{2}^{\left(B1/A1\right)}$) were screened, while 9 definite metabolites were fold >1. Other substances were indefinite or unknown. Only two definite metabolites with a greater than a 5-fold were found between NI and RI under alkali treatment, guanine (6.33-fold) and 3-phenylcatechol (−7.39-fold). Noticeably, only 8 carbohydrates, organic acids and amines (or amino acid) were 1- to 5-fold higher in RI plants than NI plants, including 3 amines [leucine (4.33-fold), O-phosphonothreonine (3.38-fold), biuret (2.23-fold)], 3 carbohydrates [fucose (1.51-fold), Glucose-1-phosphate (1.56-fold), 1,3-diaminopropane (1.71-fold)] and 2 organic acids [threonic acid (1.93-fold), 4-aminobutyric acid (1.94-fold)] (Table 1 and Table S1). Heat maps revealed organic acids in the TCA cycle and carbohydrates were higher in NI than in RI plants (Figure 6D and Table 1).

### Pathway mapping and the metabolite-to-metabolite network visualization

**Figure 7:**
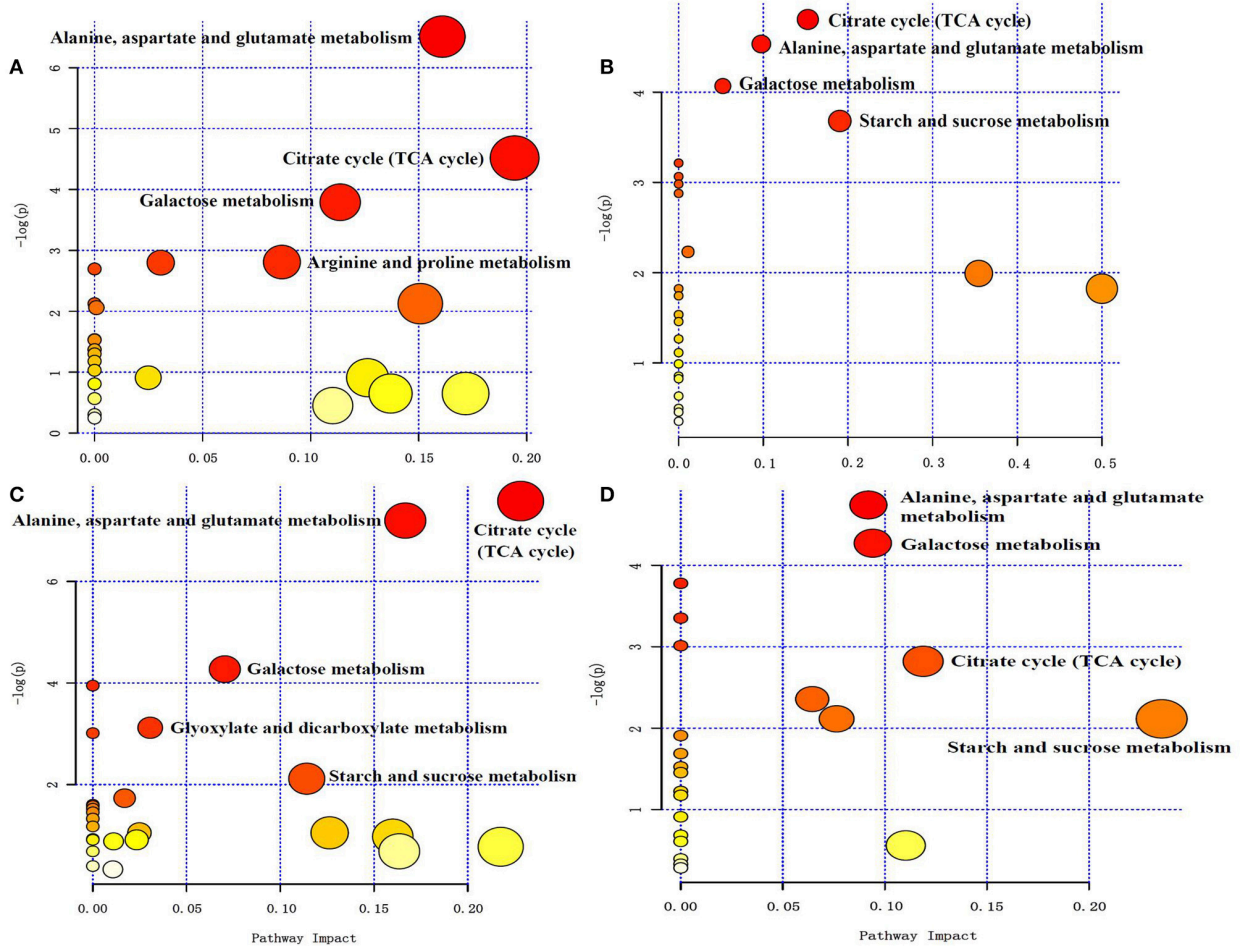
**(A)** NI Control and NI 6 days after alkali stress pathway enrichment **(B)** RI Control and RI 6 days after alkali stress pathway enrichment **(C)** NI Control and RI Control pathway enrichment **(D)** RI 6 d after alkali stress and NI 6 d after alkali stress. Pathway enrichment, the abscissa pathway impact represents the influencing factor of the path topological analysis, and the ordinate −log (P) represents the *P*-value of the pathway enrichment analysis (negative logarithm). At the same time, the bubble size indicates the influence factor of topological analysis, the bigger the bubble is, the bigger the impact factor is. The color of the bubbles indicates the *P-*value (negative logarithm) of the enrichment analysis. The darker the color, the larger the value of −log (P), the more significant the enrichment.

**Figure 8:**
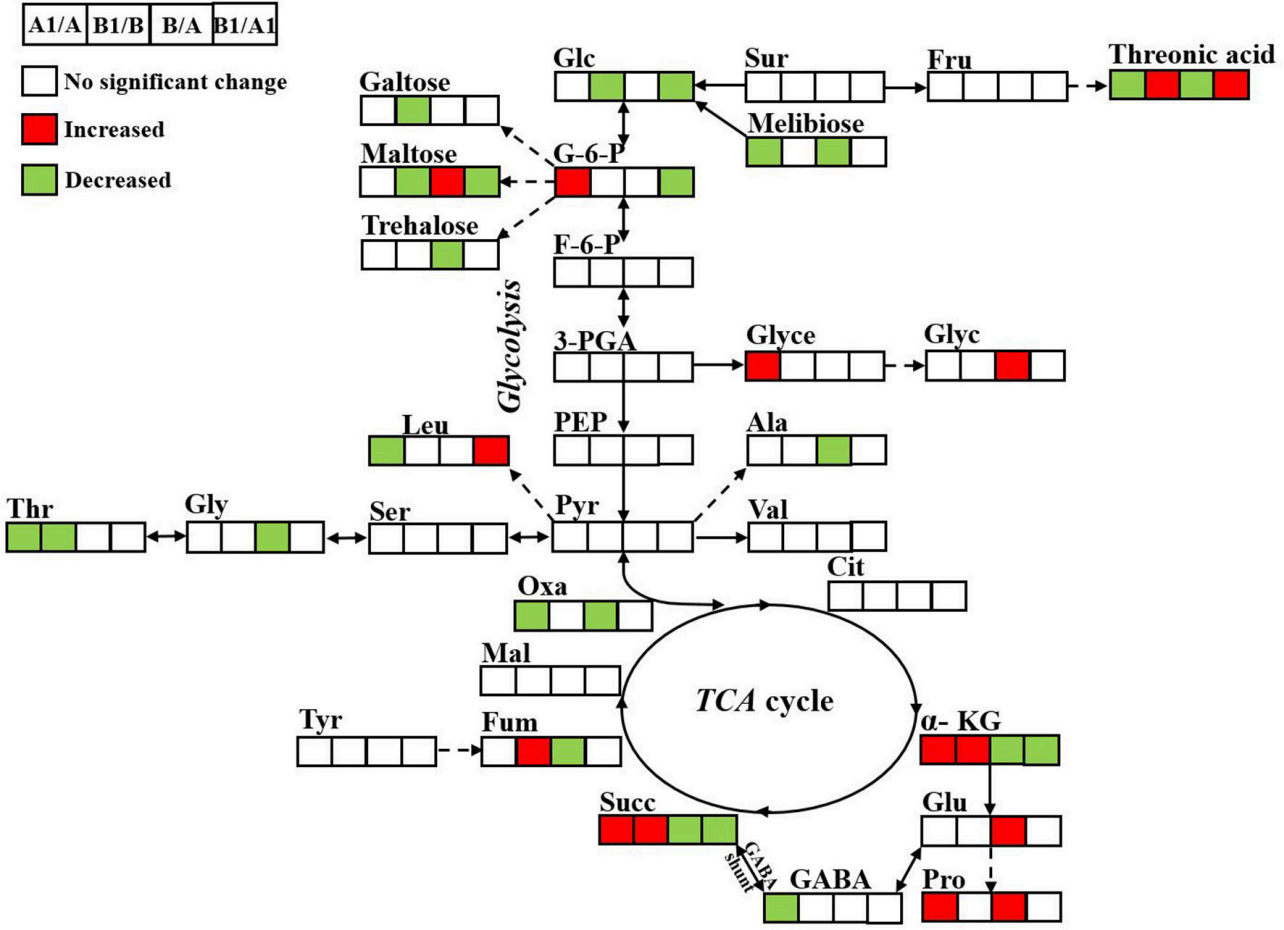
Metabolic changes involved in the primary pathways of alfalfa roots under alkali treatment. The significantly up- and down-regulated (VIP > 1, *P* < 0.05) metabolites were indicated in red and green, respectively. NI alkali-treated/NI Control (A1/A); RI alkali-treated/RI Control (B1/B), RI Control/NI Control (B/A), RI alkali-treated/NI alkali-treated (B1/A1).

All of the changed metabolites affected by symbiotic rhizobia and alkali stresses were mapped to the biological pathways involved in the KEGG database, which were assigned to 38 pathways in 4 pairwise comparisons (Table S2). The enrichment pathways were analyzed with a Bonferroni correction (*P* < 0.05 and VIP > 1). The results showed that five pathways were enriched with changed metabolites, as a result of NI and RI after alkali treatment (Figures 7A–D). Among them, three pathways, including TCA cycle (citric acid cycle), alanine, aspartate and glutamate metabolism, and galactose metabolism, were enriched for four pair-comparition groups. However, the metabolites involved in the pathway were not identical and varied in different comparisons groups, including change- fold and the trend. For example, in the galatose pathway, the fold value (${\text{log}}_{2}^{\left(A1/A\right)}$) of melibiose was −18, while in the comparition of NI- and RI- no control, the fold value was only −2.67-fold. Particularly, starch and sucrose metabolism was specific enriched in RI group, including RI- control and treatment, and NI- and RI-control, and RI- and NI-treatment. Different metabolites involving in the Starch and sucrose metabolism pathway including D-glucose, maltose, α-trehalose-6-phosphate trehalose and D-xylose. Similarly, the content of these metabolites in different samples were varies. Trehalose concentrations between the RI-and NI-control were most different (21.61-fold, fold = ${\text{log}}_{2}^{\left(NI/RI\right)}$). The altered metabolites involved in the primary metabolism of glycolysis and citric acid cycle and the linking metabolites to amino acid synthesis in alfalfa roots under alkali stress are summarized on a simplified metabolic map (Figure 8).

A schematized summary of some metabolomics variations observed between NI and RI, with or without treatment, indicated by pairwise comparisons is provided in Figure 8. Among the 20 metabolites that were differentially regulated among four pairwise comparisons, 3 were significantly different in all three comparisons, namely threonic acid, succinate, and α-ketoglutaric acid, all of which are related to amino acid metabolism and the TCA cycle.

## Discussion

Soil salinization–alkalization is one of the most serious environmental factors limit crop growth and yield (Peng et al., 2008). A better understanding of how plants cope with salt-alkali stress at the molecular and metabolic levels is required. In this study, we found that rhizobium symbiosis enhances alfalfa's resistance to alkaline stress (Figures 1, 2). A metabolomic study also revealed that a distinct metabolic profile is induced in nodulized vs. non-nodulized alfalfa (Figures 4, 5).

### Phenotypic and physiological differences between NI and RI under alkali stress

After alkali stress, alfalfa inoculated with rhizobium (RI group) showed a more resistant phenotype than non-inoculated plants (NI group). MDA content, was higher in NI than RI plants, suggesting that alkali stress-induced damage in the RI group was less than that in the NI group. On the other hand, proline was found at higher levels in RI plants than in NI plants. Proline is induced for protective purposes under stress conditions, such as drought and elevated salinity (Liu and Zhu, 1997; Delauney and Verma, 2002; Tang et al., 2014). High proline content may attenuate damage caused by osmotic stress in alkali stress.

In abiotic stress conditions, oxygen is converted to ROS, and high levels of ROS are cytotoxic (Lu et al., 2013). Under salinity stress conditions, plants increase their antioxidant levels and antioxidase activity to achieve oxidative equilibrium (Wang et al., 2001; Tang et al., 2014). Our study showed that the enzymatic activity of SOD and POD in RI plants was higher than that in NI plants, indicating that the former had a greater ROS-scavenging ability. Non-enzymatic GSH, which is an important water-soluble antioxidant and ROS scavenger, can reduce some ROS directly, thereby alleviating cellular damage due to membrane lipid peroxidization. High GSH levels increased plants' tolerance to stress and that increased GSH synthesis is an endogenous response to environmental stress (Cheng et al., 2015). However, we found that GSH was decreased greatly in RI plants 6 days after the alkali stress treatment. It is possible that GSH biosynthesis was hindered while available GSH was occupied with scavenging ROS, which is consistent with the results of Zeng and Wang (1990). Taken collectively, our analysis suggests that RI plants have a greater ROS-scavenging and oxidative stress-reducing capacity than NI plants.

### Effects of rhizobium nodulation on metabolic profile

Metabolites, which are the end products of all cellular processes and the basis for phenotype expression, can in turn affect gene transcription and protein expression. In this study, GC-TOF/MS comparative analysis was employed to identify metabolites and pathways that change after *Rhizobium* inoculation. Heat map analyses revealed metabolites with pronounced changes in association with nodulization. The substances with the most dramatic change included trehalose (decreased, −21.6-fold), 6-deoxy-D-glucose (increased, 16.9-fold), and naringin (increased, −18.5-fold). Trehalose, which is found across diverse organisms, including bacteria, yeast, and invertebrate animals (Arguelles, 2000; Chen et al., 2002), protects membranes and proteins from dehydration- and oxidation-induced damage (Rudolph and Crowe, 1985; Colaco et al., 1992). The presently observed decrease in trehalose and melibiose, following nodulation might be due to the conversion of these sugars to other sugar forms (e.g., glucose, galactose, and sucrose) to provide rhizobia with energy and carbon sources. Indeed, it has been mentioned in previous studies that trehalose is higher in symbionts (Müller et al., 1995). Other studies have shown that different species of nodule symbiosis differ in trehalose content. In this experiment, we observed lower trehalose content in the inoculated root than in normal plants; this contradiction is worthy of further study to determine the reason underlying this difference. However, as shown in Table 1, trehalose content increased more than 20-fold in the rhizobium-treated plants after 6 d of alkali stress treatment, while no such changes were observed in the non-inoculated nodulation plants. These results indicate that trehalose may be affected by alkali stress in plants with root nodule symbiosis plants. However, the existing datas could not explain this contradiction, the result was worth exploring. Sugar level changes in plants are not passive responses to stress, but rather consequent to active metabolic processes of adaption to high-pH and alkali stress (Guo et al., 2015).

Increases in amino acids, such as glutamic acid, aspartic acid, and oxoproline, might be directly related to nitrogen fixation. Organic acids serve as reductants to both the Nase complex and the respiratory chain that fuels Nase with the ATP necessary for nitrogen fixation (Kouchi and Tadakatsu, 1986; Streeter, 1987). Our observations of decreased levels of organic acids, such as succinic acid, fumaric acid, threonic acid, alpha-ketoglutaric acid, oxalacetic acid, and oxalic acid, in RI roots might be due to organic acid biosynthesis being down-regulated in response to increased root secretion of organic acids. Meanwhile, increased organic acids secretion makes the rhizosphere surrounding the root more acidic, which can neutralize alkaline salts. It is not clear why naringin levels were decreased following inoculation.

### Similarities and differences between NI and RI alfalfa in alkaline tolerance

Metabolome analysis findings indicated that the metabolite profiles of alkali-stressed NI and RI plants consisted mainly of changes in organic acid content. The accumulation of low-molecular-weight metabolites (a.k.a compatible substances) help plant cells maintain osmotic homeostasis between their cytoplasm and the environment, thus protecting normal cellular function (Bartels and Sunkar, 2005; Umezawa et al., 2006). These metabolites included nitrogen containing compounds (proline, arginine, other amino acids, quaternary ammonium compounds, and polyamines) and polyhydroxy compounds (sucrose, oligosaccharides, and polyhydric alcohols) (Bohnert et al., 1995; Hare et al., 1998).

It is notable that 4-acetamidobutyric acid, a precursor of 4-aminobutyric acid (GABA) decreased in both NI and RI plants in response to alkali stress. In plants, GABA is a stress response marker and plays an important role in both nitrogen and carbohydrate metabolism (Bouche and Fromm, 2004). The TCA cycle can engage in GABA shunting wherein various pathways bypass GABA (Fait et al., 2008). Levels of the osmoprotectant proline increase greatly after drought and salinity stress (Yang, 2012; Wang et al., 2016b). In NI plants, this increase in proline is accompanied by a significant decrease in GABA, suggesting that GABA is converted into proline to increase resistance to osmotic stress. However, in our RI group, proline levels were increased only very slightly. So, the distribution of metabolites was different between NI and RI groups after alkali stress, ultimately, the mechanism responding to alkali stress was distinctive.

Meanwhile, the increased levels of aminooxyacetic acid (AOA; an ethylene biosynthesis inhibitor) levels detected, after alkali stress suggest that ethylene biosynthesis was inhibited to increase alfalfa's tolerance to alkali stress. Experimental inhibition of ethylene biosynthesis increases plants' resistance to drought (Dezar et al., 2005; Wan et al., 2011), salinity (Achard et al., 2006; Lei et al., 2011), and cold (Shi et al., 2012). On the other hand, augmentation of ethylene content in wild *Arabidopsis* plants with an ethylene biosynthesis precursor (i.e., 1-aminocyclopropane-1-carboxylic acid) enhanced resistance to high salinity conditions and survival (Cao et al., 2007). Thus, although ethylene appears to somehow be linked to stress responses in plants, the mechanisms of this linkage are unknown. Moreover, the influence of ethylene on stress resistance may differ across genera.

The differing metabolite shifts observed following alkali stress treatment in NI plants vs. RI plants in our study suggest that these two groups may have undergone different resistance mechanisms. We hypothesize that the increased organic acid production in RI plants may reflect adaptive strategies for maintenance of cellular homeostasis. Organic acid accumulation in vacuoles affects cellular pH and reduces excessive cations (Shi and Zhao, 1997; Yang et al., 2007; Guo et al., 2013). The pronounced accumulation of secondary metabolites in severely wilting and growth-stunted NI plants 6 days after the alkali stress treatment may be due to the degradation of macromolecules. Plants absorb ${\text{NO}}_{3}^{-}$ from soil, which is converted into ${\text{NO}}_{2}^{-}$ by nitrate reductase. The ${\text{NO}}_{2}^{-}$ is then converted into ${\text{NH}}_{4}^{+}$ by nitrite reductase. The ${\text{NO}}_{3}^{-}$ content in alfalfa and wheat roots is near zero under alkali stress (Wang et al., 2012; Guo et al., 2015). The enzyme activities of nitrate reductase and glutamine synthetase, which are induced by ${\text{NO}}_{3}^{-}$, decrease in the absence of ${\text{NO}}_{3}^{-}$. Consequently, amino acid biosynthesis is inhibited, which in turn affects almost all aspects of metabolism. Hence, greatly reduced ${\text{NO}}_{3}^{-}$ content in NI plants may have affected a variety of metabolism processes. It is our view that the symbiotic nodules in RI plants may have compensated for reduced ${\text{NO}}_{3}^{-}$, thereby reducing damage and conferring enhanced resistance.

Taken collectively, our data indicate that *Rhizobium* symbiosis increases alfalfa's resistance to alkali stress. The physiologic mechanisms of enhanced resistance may be associated with increased antioxidant activity (via MDA, POD, SOD, and GSH), enhanced production of organic acids, and accumulation of osmolytes such as proline. In metabolomics levels, different metabolites were enriched in TCA cycle (citric acid cycle), alanine, aspartate and glutamate metabolism, and galactose metabolism for four pair-comparition groups. However, the metabolites were not identical and varied in different comparisons groups. RI and NI groups had different distribution of metabolites, and distinctive response after alkali stress. For RI group, the accumulation of organic acids, amino acids and secondary metabolites in the process of C-N transformation had played a positive role in promoting alkali resistance.

## Author contributions

HC and GC contributed to the conception of the study. TS and HX contributed significantly to analysis and manuscript preparation. TS performed the data analyses and wrote the manuscript. NS, LJ, PT, YY, and WY helped perform the analysis with constructive discussions.

### Conflict of interest statement

The authors declare that the research was conducted in the absence of any commercial or financial relationships that could be construed as a potential conflict of interest.

## Supplementary material

Figure S1 Plot of R2 and Q2 values from 200 permutations. Green circle, R2; blue square, Q2. The green and blue lines represent the regression lines for R2 and Q2, respectively. **(A)** NI Control and NI alkali-treated **(B)** RI Control and RI alkali-treated **(C)** NI Control and RI Control **(D)** NI alkali-treated and RI alkali-treated.

### FootNotes

^1^Nikkaji: http://jglobal.jst.go.jp/en/

^2^ChEBI: http://www.ebi.ac.uk/chebi/init.do

^3^PubChem: https://pubchem.ncbi.nlm.nih.gov

^4^CAS: http://www.anychem.com

^5^KEGG: http://www.genome.jp/kegg/

^6^HMDB: http://www.hmdb.ca
