# Supplementary figures and images for: Metabolomic Analysis of Alfalfa (Medicago sativa L.) Root-Symbiotic Rhizobia Responses under Alkali Stress
Source: Front Plant Sci. 2017 Jul 11;8:1208. doi: 10.3389/fpls.2017.01208 (PMC5504246; doi:10.3389/fpls.2017.01208)

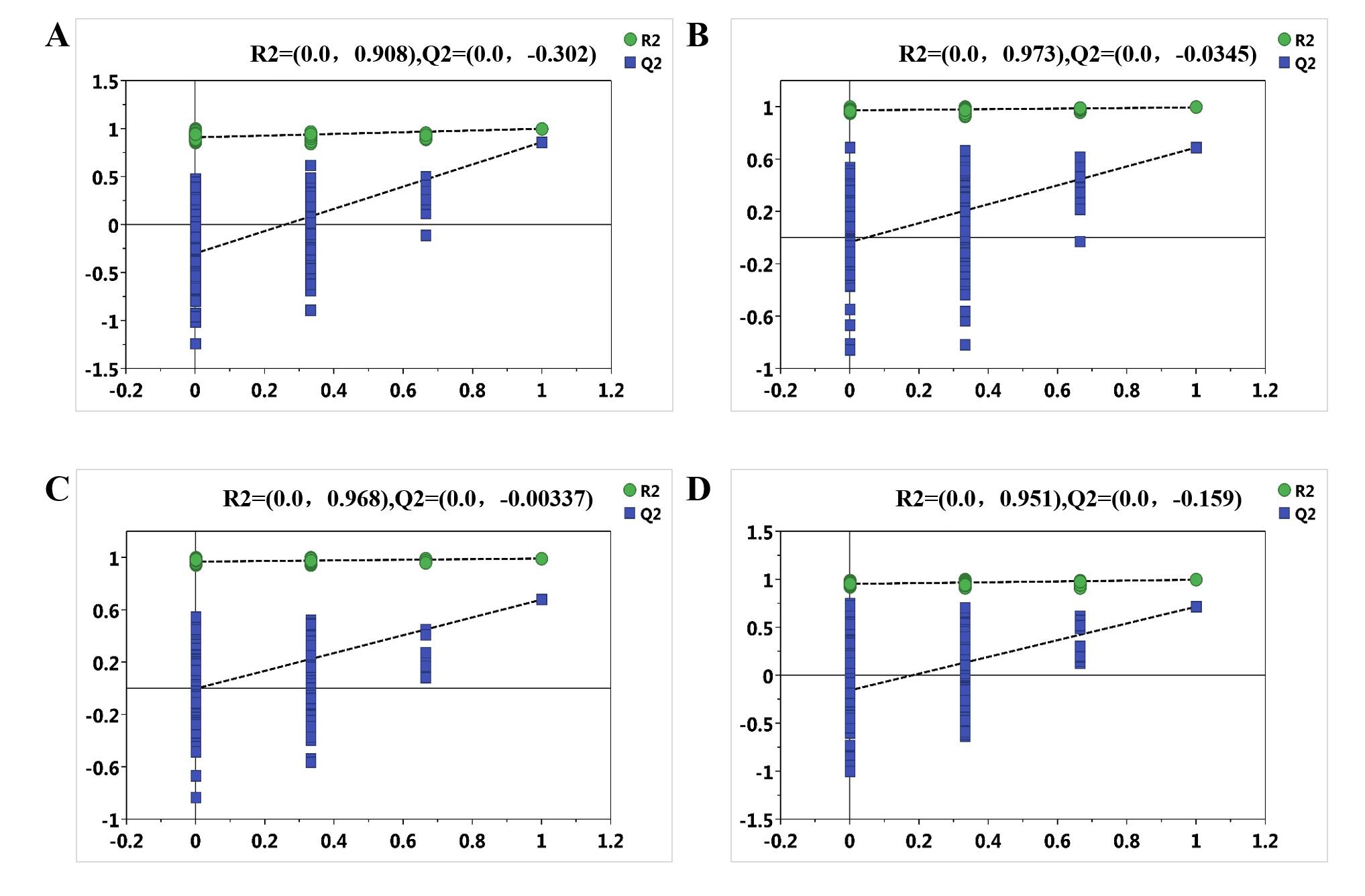

Supplement: Figure S1 — Plot of R2 and Q2 values from 200 permutations. Green circle, R2; blue square, Q2. The green and blue lines represent the regression lines for R2 and Q2, respectively. (A) NI Control and NI alkali-treated (B) RI Control and RI alkali-treated (C) NI Control and RI Control (D) NI alkali-treated and RI alkali-treated. [file Image1.JPEG]
